# Nasal colonization and bacterial contamination of mobile phones carried by medical staff in the operating room

**DOI:** 10.1371/journal.pone.0175811

**Published:** 2017-05-31

**Authors:** Chih-Hsiang Chang, Szu-Yuan Chen, Jang-Jih Lu, Chee-Jen Chang, Yuhan Chang, Pang-Hsin Hsieh

**Affiliations:** 1 Department of Orthopedic Surgery, Chang Gung Memorial Hospital, Linkou, Taiwan; 2 College of Medicine, Chang Gung University, Taoyuan, Taiwan; 3 Bone and Joint Research Center, Chang Gung Memorial Hospital, Linkou, Taiwan; 4 Graduate Institute of Clinical Medical Sciences, College of Medicine, Chang Gung University, Taoyuan, Taiwan; 5 Department of Medical Biotechnology and Laboratory Science, College of Medicine, Chang Gung University, Taoyuan, Taiwan; 6 Department of Laboratory Medicine, Chang Gung Memorial Hospital, Linkou, Taoyuan, Taiwan; 7 Research Services Center for Health Information, Chang Gung University, Taoyuan, Taiwan; 8 Clinical Informatics and Medical Statistics Research Center, Chang Gung University, Taoyuan, Taiwan; Leibniz-Institute DSMZ, GERMANY

## Abstract

**Background:**

Mobile phones (MPs) have been an essential part of the lives of healthcare professionals and have improved communication, collaboration, and sharing of information. Nonetheless, the widespread use of MPs in hospitals has raised concerns of nosocomial infections, especially in areas requiring the highest hygienic standards such as operating rooms (ORs). This study evaluated the incidence of bacterial contamination of the MPs carried by medical staff working in the OR and determined its association with bacterial colonization of this personnel.

**Methods:**

This is an observational cohort study. Medical staffs working in the OR were asked to take bacterial cultures from their MPs, anterior nares, and dominant hands. To identify the relation between MP contamination and bacterial colonization of the medical staff, genotyping of *Staphylococcus aureus* (SA) was done via *Staphylococcus* protein A gene (*spa*) typing and pulsed-field gel electrophoresis (PFGE).

**Results:**

A total of 216 swab samples taken from 72 medical-staff members were analyzed. The culture-positive rate was 98.1% (212/216). In 59 (27.3%) samples, the bacteria were possible clinical pathogens. The anterior nares were the most common site of colonization by clinical pathogens (58.3%, 42/72), followed by MPs (13.9%, 10/72) and the dominant hand (9.7%, 7/72). SA was the most commonly isolated clinical pathogen and was found in 43 (19.9%) samples. In 66 (94.3%) of the 70 staff members for whom bacteria were detected on their MPs, the same bacteria were detected in nares or hand. Among 31 medical staff who were carriers of SA in the anterior nares or dominant hand, 8 (25.8%) were found to have SA on their MPs, and genotyping confirmed the same SA strain in 7 (87.5%) of them.

**Conclusion:**

A high rate of bacterial nasal colonization and MPs contamination were found among the OR medical staff. An MP may be a reservoir for pathogen contamination in the OR.

## Introduction

Hospital-acquired infection (HAI) is a serious problem in hospitals and may cause increased morbidity and mortality [1]. It is estimated that between 5% and 10% of hospitalized patients have HAI, and recent data suggest that this figure is on the rise [2]. In the United States, the reported cost of HAI in 2002 was $6.7 billion [3], and increased to 2 million cases/year, 100,000 deaths, and costs $20 billion yearly [4]. As early as 1861, Semmelweis showed that bacteria are transmitted to patients via the contaminated hand of healthcare workers [5]. The transmission of HAI occurs through hospital staff and via the animate hospital environment, equipment, and devices that the hospital staff use [6].

Mobile phones (MPs) have become an integral part of the lives of healthcare professionals and have improved communication, collaboration, and sharing of information. Widespread use of MPs in hospitals has raised concerns of nosocomial infections, especially in areas requiring the highest hygienic standards such as the operating room (OR). In other studies, prevalence of MPs contaminated with bacteria was reported to range from 62% to 99% [7–10], and potential clinical pathogens were detected up to 14.3–75% MPs [7, 8, 10–13]. Brady et al. [11] found that 14.3% of MPs are colonized by bacteria known to cause nosocomial infections. Khivsara et al. even found that there is a 40% rate of MSSA (6.7% MRSA) colonization of MPs [14]. Borer et al. [15] found MPs to be an important cause of HAI.

*Staphylococcus aureus* (SA) is a common pathogen in osteomyelitis [16] and periprosthetic joint infection [17]. Patients who are colonized with SA are at a 2- to 9-fold higher risk of SA infection [18]. The most common area for SA colonization in the human body is nasal nares [19], and high prevalence of contamination of MPs was also reported [7–11, 14]. In public opinion, hospital OR should be the workplace with the highest hygienic standards. The same high hygienic requirements also hold for the personnel working there and the equipment used by them. Nonetheless, MPs are not forbidden in the OR [20].

Nowadays almost everyone has at least one mobile device and carry them anytime and virtually everywhere. In the OR, medical staffs are asked to change surgical gowns and wear a surgical mask at all times to prevent from spreading bacteria to the environment. The use of personal MPs, however, has not been restricted in the OR mostly. Contamination of MPs revealed a potential bacteria reservation and a potential source of bacterial spreading in the OR. The colonization of medical staff’s nasal nares may be transmitted to MPs by hands. The relation among the rates of colonization of medial staff’s nares, hands, and MPs is currently unclear. Are hands randomly contaminated via MPs, or contaminated from a user’s nasal colonization? The purposes of this study were to evaluate the colonization status of orthopedic medical staff working in an OR in terms of nasal nares, hands, and MPs, and to identify the bacteria involved. The SA that was isolated was chosen for further genotyping to clarify the bacterial transmission among nares, hands, and MPs. We hypothesized that nasal colonization by SA may be transmitted to hands and MPs.

## Materials and methods

### Sample collection and processing and isolation of microorganisms

This is an observational cohort study. After approval by the Institutional Review Board of Chang Gung Memorial Hospital (IRB approval No. 102-4862B), the study was initiated in January 2014 and ended in March 2014. The medical staff working in the OR was enrolled after written informed consent was obtained. In the Department of Orthopedic Surgery, there were totally 112 medical-staff who worked in the OR, including 60 OR nurses and 62 surgeons. We excluded 21 staffs who were in night shifts, 9 who were on vacation and 10 who were not willing to participate the study, leaving 72 in the analysis. The characteristics were listed in Table 1.

**Table 1:** Characteristics of medical-staff.

	Participate	Total
Age	37.60 ipater	38.95 ipater
Sex M/F	46/26	64/36
Nurse	19	41
Nurse partitian	6	9
Resident	22	24
Attending doctor	25	38
	72	112

During daily work in the OR, samples were taken between every operation, 30 minutes after removing surgical groove at the end of operation. Samples for bacterial culture from the anterior nares, dominant hands, and MPs were collected with a cotton swab moistened with distilled water. The MPs belonged to the medical staffs and were used only by owners. Culture samples from anterior nasal nares were collected by rubbing the swab against the anterior 1 cm of the nasal vestibular wall of both nares. After swabbing around anterior nasal nares, dominant hand, or mobile phone respectively, a culture plate was streaked with the cotton swab immediately in the OR. The cotton swab of every sample (anterior nares, dominant hands, or MPs) was cultured on BP/EMB (blood agar plate/eosin methylene blue agar plate) and CNA (Columbia-colistin and nalidixic acid agar) on one fourth of the plate, and then we snapped the tip of the cotton swab into Thioglycollate broth without contamination of surrounding objects.

During sampling, the MP was swabbed all around. The dominant hand was swabbed both dorsally and ventrally and between the fingers. The nasal nares were swabbed the swab against the anterior 1 cm of the nasal vestibular wall of both nares. The culture plate and broth were transported to microbiology laboratory after the above procedure immediately. After the plate was delivered to the microbiology laboratory, the plate was streaked with a sterilized iron stick over the remaining three-fourths of the plate in a cell culture hood. Then, the culture plate and broth were incubated at 37°C for 48 h. In every time slot (every 2 h) during sampling in the OR, a negative control was set up as follows: the tip of a cotton swab moistened with distilled water was snapped into Thioglycollate broth.

### Identification of the microorganisms

Isolated microorganisms were identified using the Gram stain, pigmentation, colony morphology, catalase assay, motility, esculin hydrolysis, Staphaurex Plus (Remel Europa Ltd., Dartford, UK), and Api-System (bioMerieux, Marcy-l’Etoile, France). The bacteria isolated as coagulation-negative *Staphylococcus* (CNS), gram-positive bacillus (GPB), *Streptococcus* spp. (Strep), *Moraxella* spp., and gram-negative *Bacillus* (GNB) were considered as normal flora [21]. The isolated bacteria were subjected to matrix-assisted laser desorption ionization time-of-flight mass spectrometry (MALDI-TOF MS) [22] for confirmation of the identification. If the culture revealed SA, further genotyping by means of surface protein A (*spa*) and pulsed-field gel electrophoresis (PFGE) were performed to identify the strain.

### Genotyping

#### DNA extraction

Whole-cell (genomic) DNA, used as a template for PCR amplification, was prepared from single colonies using the Wizard^®^ Genomic DNA Purification Kit (Promega, Dübendorf, Switzerland). The PCR products were purified for sequencing using the PureLink^™^ Quick Gel Extraction and PCR Purification Combo Kit (Invitrogen, Zug, Switzerland).

#### Spa typing

The polymorphic repeat region (region x) of the SA protein A encoded by the *spa* gene was amplified as described previously [23] using primers listed below. Using a software package (Ridom StaphType software version 1.4, Ridom GmbH, Wurzburg, Germany), the *spa* types of SA strains were analyzed and the new *spa* type assignment was provided automatically through the Ridom SpaServer (http://spa.ridom.de/index/shtml). The BURP (Based Upon Repeat Patterns) can be used to assign *spa* clonal complexes (CCs).

#### SA protein A primers

1095F AGACGATCCTTCGGTGAGC

1517R GCTTTTGCAATGTCATTTACTG[23]

#### Pulsed-field gel electrophoresis (PFGE)

The DNA fingerprints generated by PFGE were analyzed by both the manual method according to the criteria proposed by Tenover et al. [24] and by a digitized method using BioNumerics Fingerprint types and Cluster Analysis software (Applied Maths, Austin, TX). Percent similarities were identified on a dendrogram built by the unweighted pair group method using arithmetic averages and Dice coefficients. Band position tolerance and optimization were set to 1.25 and 0.5%, respectively.

## Results

There were 216 swab samples taken from 72 medical-staff members included in the study, with 3 samples (nares, hands, MPs) per person. The overall bacteria-positive rate was 98.1% (212/216), the highest in nasal nares (100%, 72/72), followed by dominant hands (97.2%, 70/72) and MPs (97.2%, 70/72). The isolated microorganism was a possible clinical pathogen in 27.3% (59/216) of the samples, and was most frequently found in nasal nares (58.3%, 42/72), followed by MPs (13.9%, 10/72) and hands (9.7%, 7/72; Table 2). The most common clinical pathogen was SA (19.9%, 43/216), with 27 methicillin-sensitive strains (MSSA, 12.5%, 27/216) and 16 methicillin-resistant strains (MRSA, 7.4%, 16/216), followed by *Enterobacter* spp. (5.6%, 12/216) and *Citrobacter koseri* (4.6%, 10/216; Table 3).

**Table 2:** Isolation propotion in different culture.

Nasal nares	Culture positive	100%(72/72)	Possible clinical pathogen	58.33%(42/72)
			Normal flora	100%(72/72)
	Culture negative	0		
Hands	Culture positive	97.2%(70/72)	Possible clinical pathogen	9.72%(7/72)
			Normal flora	97.2%(70/72)
	Culture negative	2.8%(2/72)		
MPs	Culture position	97.2%(70/72)	Possible clinical pathogen	13.89% (10/72)
			Normal flora	97.2%(70/72)
	Culture negative	2.8%(2/72)		

MPs: Mobile phones

**Table 3:** Distribution of isolated bacteria.

	Nasal Nares	Hands	MPs
Isolation rate	100% (72/72)	97.20% (70/72)	97.20% (70/72)
**Possible clinical pathogen**			
**Gram-positive**			
MSSA	21	1	5
MRSA	10	3	3
**Gram-negative**			
*Enterobacter aerogenes*	10	1	1
*Escherichia coli*	1		
*Acinetobacter baumannii*	1	3	1
*Klebsiella pneumoniae*	1		
*Pseudomonas putida*			2
*Citrobacter koseri*	10		
**Normal flora**			
CNS	70	60	65
GPB	35	24	14
*Streptococcus* spp.	5	13	10
*Moraxella* spp.		3	2
GNB			2

MSSA: Methicillin-sensitive *Staphylococcus aureus*

MRSA: Methicillin-resistant *Staphylococcus aureus*

CNS: Coagulation-negative *Staphylococcus*

GPB: Gram-positive *Bacillus*

GNB: Gram-negative *Bacillus*

In 72 medical-staff members, there were 70 people having a positive culture from their MPs (97.2%, 70/72). Among that, 66 (94.3%, 66/70) were found to have the same microorganism in the nares or hands (nares only, 16.7%; hands only, 4.5%; both, 78.8%). Among the 12 participants for whom a clinical pathogen was isolated from their MPs, the same clinical pathogen was also found in 10 participants (83.3%) in their nares or dominant hands (nares, 70%; hands, 10%; both, 20%).

The distribution of SA discovered at the 3 sites is shown in Table 4. SA was found in 31 (31/72; 43%) swab samples from anterior nares, 8 samples (8/72; 11.1%) from MPs, and 4 (4/72; 5.6%) from the hands. Among these samples, methicillin-resistant strains were found in 10 (32.3%), 3 (37.5%), and 3 (75%) samples from the anterior nares, MPs, and hands, respectively. It should be noted that among 31 SA carriers, 8 (25.8%) had growth of SA in cultures from their MPs, and all 8 medical staff who had SA in cultures from their MPs were SA carriers (6 in the anterior nares and 2 in the anterior nares and hand). Genotyping using Spa and PFGE confirmed the same strains of SA in 7 of them (87.5%, 7/8).

**Table 4:** Distribution and genotyping of *Staphylococcus aureus*.

Staff No	Drug sensitivity	Nasal Nares		Hands		MPs	
		Spa	PFGE	Spa	PFGE	Spa	PFGE
*1	S	S1	I			S1	I
6	S	S2	A				
*8	R	t4911	E	t4911	E	t4911	E
9	S	t1209	A				
*10	R	t026	B	t026	B		
11	S	t4911	D				
13	S	S3	H				
*15	S	S4	F			S4	F
19	S	S5	F				
*21	R/S	t026	C			S6	J
24	R	S7	L				
*29	R	t1751	G			t1751	G
32	S	S8	A1				
33	S	S9	K				
37	S	t213	Q				
39	R	t4911	X				
41	S	t2379	R				
42	S	t5353	W				
*48	S	S10	M	S10	M		
49	S	t338	N				
51	S	t5693	O				
52	S	t189	P				
*53	R	t441	S	t441	S	t441	S
55	S	t5693	T				
58	S	S11	U				
*61	S	S12	V			S12	V
62	R	t4911	Y				
66	S	t737	AA				
67	R	t437	AB				
68	R	t437	AC				
*71	S	t2868	AD			t2868	AD

* *Staphylococcus aureus* isolation from both nares and either dominant hand or mobile phone

S1–S12: Repeat succession showed in S1 File

MPs: Mobile phones

*Spa*: Staphylococcus protein A gene

PFGE: Pulsed-field gel electrophoresis

S: sensitive

R: resistant

## Discussion

MPs contaminated with bacteria had been studied well [7–14], but the relation between nasal colonization and contamination of MPs is not well understood. For further evaluation of this relation, we initiated this study by doing culture analysis of nares, hand, and MP. Further SA genotyping with *spa* analysis and PFGE was also conducted to clarify the relation among nasal, hands, and MPs colonization proportion.

A high identification proportion, up to 98.1% (212/216), was observed in our study. Review of other studies suggests that the contamination rates of MPs vary from 62% to 90% [7–10], and detection of potential clinical pathogens also varies: from 14.3% to 75% of samples [7, 8, 10–13]. The reason for the large variation may be the sampling from different types of MPs owners (healthcare workers, anesthesiologists, or doctors at a teaching hospital), sampling methods (cotton swab was moistened or not before sampling, immediate streaking of the plate or not, final placement of the cotton swab tip into culture broth or not), and where the culture plate was streaked with the cotton swab (in room air, in a cell culture hood after transportation to a microbiology laboratory, or in the OR under laminar flow conditions). There are only a few papers with a high identification rate, up to 90% [9, 10], and those authors used moistened cotton swabs to obtain the samples. We tried to optimize the culture rate and lower the possible contamination by using moistened cotton swabs, immediate streaking on the culture plate under OR laminar flow conditions (transported the material to a microbiology laboratory to streak the rest of the culture plate), and placed the cotton swab tip into culture broth for enriched culture finally. The sampling was done in nares, dominant hands, and MPs at the same time for us to trace the possible transmission. The distribution of bacteria among nares, hands, and MPs was reasonable, with the biggest presence in nares, followed by MPs and hands (Tables 2 and 3). The nares are a moist and warm environment for bacterial colonization; this situation probably contributed to the highest isolation rate. MPs are rarely cleaned up, with a little bit higher isolation rate than from hands, which are frequently washed in the OR. It was warning that nasal colonization might be passed on to MPs by hands.

After we reviewed the results of bacterial isolation, the majority of bacteria were CNS. The CNS colonization rate was up to 97.2% (70/72) in nasal nares, followed by MPs (90.2%, 65/72) and hands (83.3%, 60/72; Table 3), comparable with data from other studies—by Ulger et al. [9] and Pal et al. [25]—who showed that the most common isolated bacterium from hands and MPs is CNS. The potential clinical pathogens identified in this study are a cause for concern. Among the medical staff, 58.3% members (42/72) carried potential clinical pathogens, and most of these bacteria were isolated from nares (Table 3). Among possible clinical pathogens, the most common species was SA. There were 43% (31/72) SA and 13.9% (10/72) MRSA cases among swab samples from nares (Table 3). According to other studies, the nasal colonization rate among hospital janitors is 15.3% [26], and the nasal colonization rate among residents in a nursing home is 18.3% [27]. The highest colonization rate was reported among patients in an Indian hospital: up to 37% [28]. In Taiwan, studies revealed that nasal MRSA colonization rates among healthcare workers (5.0–7.8%) [29] and pediatricians (6.8%) [30] are significantly higher than the rate in the general population (3.8%) [29–31]. If we compare our results with data from other studies, the SA nasal colonization among our medical staff was much higher, even higher than among patients. The possible reason is the methods of sample collection as already mentioned, and our method allowed us to trace the transmission on the basis of the high SA isolation rate. Nevertheless, not only was the nasal colonization rate of SA high, the prevalence of MRSA was high (32.3% of SA-positive samples) too. Even in comparison with a recent study involving sampling from pediatricians and polymerase chain reaction (PCR) analysis [30], our MRSA isolation rate is still higher (13.9% versus 6.8%). Because of the high colonization rate in nares with high prevalence of resistant strains, additional measures for intraoperative sterilization and avoidance of contamination can be recommended.

As the most popular nonmedical electronic equipment, MPs are used in close contact with the body and often without cleaning guidelines that meet hospital standards. The bacterium most frequently isolated from MPs in our study is CNS, followed by *Streptococcus* spp. and SA. There was also a gram-negative bacterium (11.1%, 8/72). The nasal colonization of medical staff may be transmitted to patients via hands, but there is no evidence to prove that yet. For all bacteria, isolation from MPs coincided with isolation from nares or hands in 94.3% of such cases (66/70). Among 31 medical staff for whom SA was isolated from their MPs, anterior nares, or dominant hands, 8 (25.8%) were found to have SA on MPs. As the most common pathogen in human bodies causing tissue infections, pneumonia, septicemia, and device-associated infection, especially in the orthopedic field including osteomyelitis [16] and periprosthetic joint infection [17], SA was chosen for genotyping to confirm the transmission pattern. For 8 SA-positive MPs, the same SA strain was found in 7 samples from nares (87.5%); this finding was proved by *spa* and PFGE typing (Table 4). Among those 7 SA-positive cultures, 3 were MRSA, and 4 were MSSA. MRSA seemed to be transmitted to MPs more easily than MSSA did (33.3% vs. 19.0%, 3/9 vs. 4/21; Table 4).

We chose 2 types of genotyping to confirm the transmission of SA: PFGE and SA A gene (*spa*). PFGE is the gold standard of SA strain typing although it is time-consuming and the interlaboratory comparability of results requires extensive effort by harmonization of protocols. To make genotyping easier (sequence data can be transferred between laboratories via the Internet), DNA sequence-based approaches are becoming more popular. There is evidence for recombination in SA, where single-locus DNA sequencing of repeat regions of the coagulase gene (*coa*) and the *spa* gene can be used for reliable and accurate typing of SA [32, 33]. The *spa* typing is especially valuable for rapid typing of SA in hospital settings because it offers higher resolution than *coa* typing. The *spa* typing was not commonly used in the past because of problems with a consensus on assignment of new *spa* repeats and types. Nevertheless, software Ridom StaphType was developed which now synchronizes data either directly via the http protocol or via a file-based transfer (e.g., by e-mail) with an accompanying SpaServer that functions as an operative source for all new *spa* repeats and type codes [34]. We chose PFGE and *spa* typing for every strain of SA to reliably identify SA transmission among nares, hands, and MPs.

We tried to evaluate the colonization status of orthopedic medical staff working in an OR by identifying the bacterial flora with further genotyping of SA to clarify the relation among nasal, hands, and MPs colonization rates. However, there are some limitations to this study. First, we performed genotyping of only SA to trace and confirm the transmission from nares to hands and MPs. The transmission of other bacteria may not be the same as that for SA. Nevertheless, SA was found to be the most common isolated bacterium among possible clinical pathogens for us to trace. Second, normal flora in this study was defined according to a definition of specific types of infections [21]. On the other hand, normal flora as defined here may be potentially infective. For example, CNS here was identified as normal flora. Nowadays, CNS is classified into several strains [35], some of which were reported to cause a disease, such as *Staphylococcus epidermidis [36]* and *Staphylococcus capitis [37]* reported as an opportunistic human pathogen. The majority of the bacterial isolates in this study were CNS: a cause for concern. Third, although we confirmed the transmission of nasal colonization to hand and MP by genotyping, we could not identify a direct relation between colonization of medical staff and surgical-site infections (SSIs). In our hospital, the definition of SSI is that infection was noticed by symptoms (ecchymosis, swelling, local heat, or tenderness) or pus formation proved by tapping or imaging study within 1 year after surgery. And The incidence of SSIs in our hospital in recent 4 years was around 0.3~0.8%, with 30.2% of MSSA, 12.7% of MRSA, 4.8% CoNS, and 15.9% Gram-negative bacteria. Although the pathogens of SSIs were similar to the bacteria isolated in this study, further studies on bacteria isolated from infection sites and from colonized medical staff or patients are needed to clarify this issue.

In conclusion, at a high isolation rate of bacteria, the same bacteria in MPs and nares or hands were often detected here (94.3%, 66/70). The high rate of SA nasal colonization (43.1%, 31/72) was observed, and in 22.6% of cases (7/31), this colonization may be passed on to MPs according to *spa* and PFGE genotyping confirmation. The same SA strain on MPs and nares or hands was also proved in most cases (87.5%, 7/8). As for SA contamination, MRSA was transmitted more easily from nares to hands or MPs than MSSA was (33.3% vs. 19.0%). Therefore, MPs may serve as a reservoir of potential pathogens in the OR from a user’s nasal colonization because the same strain was confirmed by *spa* and PFGE genotyping. As the pathogen of SSIs was similar to contamination pathogens on MPs, and transmission of bacteria was probed by genotyping, further research about the relationship between SSIs and MPs are needed. With the evidence revealed in this study, we suggested that the use of personal MPs sholud be regulated in the ORs. If possible, regular clean-up is suggested for decreasing MPs contamination.

## Supporting information

S1 File Repeat succession of *Staphylococcus aureus*. (DOCX)

## References

[1] Plowman R, Graves N, Griffin MA, Roberts JA, Swan AV, Cookson B, et al The rate and cost of hospital-acquired infections occurring in patients admitted to selected specialties of a district general hospital in England and the national burden imposed. J Hosp Infect. 2001;47(3):198–209. 10.1053/jhin.2000.0881 11247680

[2] Hacek DM, Suriano T, Noskin GA, Kruszynski J, Reisberg B, Peterson LR. Medical and economic benefit of a comprehensive infection control program that includes routine determination of microbial clonality. Am J Clin Pathol. 1999;111(5):647–54. 10230355 10.1093/ajcp/111.5.647

[3] Haley RW. Incidence and nature of endemic and epidemic nosocomial infections In: Bennett JV, Bracham P, editors Hospital infections Boston: Little Brown 1985:359–74.

[4] Sprague L. Health care-associated infections: is there an end in sight? Issue Brief Natl Health Policy Forum. 2009;(830):1–14. 19343832

[5] Semmelweis I. Die aetiologie, der begriff, und die prophylaxis des kindbettfiebers. Pest, Wien u Leipzig, CA Hartleben. 1861.

[6] Kolmos HJ. [Hospital infections: sources and routes of infection]. Ugeskr Laeger. 2007;169(48):4138–42. 18211776

[7] Chawla K MC, Gurung B, Bhate P, Bairy I. Bacterial ‘cell’ Phones: Do cell phones carry potential pathogens? Online J Health Allied Scs. 2009;(8):8.

[8] Akinyemi KO, Atapu AD, Adetona OO, Coker AO. The potential role of mobile phones in the spread of bacterial infections. J Infect Dev Ctries. 2009;3(8):628–32. 19801807 10.3855/jidc.556

[9] Ulger F, Esen S, Dilek A, Yanik K, Gunaydin M, Leblebicioglu H. Are we aware how contaminated our mobile phones with nosocomial pathogens? Ann Clin Microbiol Antimicrob. 2009;8:7 10.1186/1476-0711-8-7 19267892 PMC2655280

[10] Bhat SS HS, Salian S. Potential of mobile phones to serve as a reservoir in spread of nosocomial pathogens. Online J Health Allied Scs. 2011;(10):14.

[11] Brady RR, Wasson A, Stirling I, McAllister C, Damani NN. Is your phone bugged? The incidence of bacteria known to cause nosocomial infection on healthcare workers' mobile phones. J Hosp Infect. 2006;62(1):123–5. 10.1016/j.jhin.2005.05.005 16099536

[12] Sadat-Ali M, Al-Omran AK, Azam Q, Bukari H, Al-Zahrani AJ, Al-Turki RA, et al Bacterial flora on cell phones of health care providers in a teaching institution. Am J Infect Control. 2010;38(5):404–5. 10.1016/j.ajic.2009.08.007 20363049

[13] Kilic IH, Ozaslan M, Karagoz ID, Zer Y, Davutoglu V. The microbial colonisation of mobile phone used by healthcare staffs. Pak J Biol Sci. 2009;12(11):882–4. 19803124 10.3923/pjbs.2009.882.884

[14] Khivsara A ST, Dahashree B. Typing of Staphylococcus aureus from mobile phones and clinical samples. Current science. 2006;(90):910–2.

[15] Borer A, Gilad J, Smolyakov R, Eskira S, Peled N, Porat N, et al Cell phones and Acinetobacter transmission. Emerg Infect Dis. 2005;11(7):1160–1. 10.3201/eid1107.050221 16032803 PMC3371817

[16] Russell CD, Ramaesh R, Kalima P, Murray A, Gaston MS. Microbiological characteristics of acute osteoarticular infections in children. J Med Microbiol. 2015;64(Pt 4):446–53. 10.1099/jmm.0.000026 25596125

[17] Chen SY, Hu CC, Chen CC, Chang YH, Hsieh PH. Two-Stage Revision Arthroplasty for Periprosthetic Hip Infection: Mean Follow-Up of Ten Years. Biomed Res Int. 2015;2015:345475 10.1155/2015/345475 26064901 PMC4429212

[18] Wenzel RP, Perl TM. The significance of nasal carriage of Staphylococcus aureus and the incidence of postoperative wound infection. J Hosp Infect. 1995;31(1):13–24. 7499817 10.1016/0195-6701(95)90079-9

[19] Hadley S, Immerman I, Hutzler L, Slover J, Bosco J. Staphylococcus aureus Decolonization Protocol Decreases Surgical Site Infections for Total Joint Replacement. Arthritis. 2010;2010:924518 10.1155/2010/924518 22046511 PMC3200003

[20] Klein AA, Djaiani GN. Mobile phones in the hospital—past, present and future. Anaesthesia. 2003;58(4):353–7. 12648117 10.1046/j.1365-2044.2003.03079.x

[21] Horan TC, Andrus M, Dudeck MA. CDC/NHSN surveillance definition of health care-associated infection and criteria for specific types of infections in the acute care setting. Am J Infect Control. 2008;36(5):309–32. 10.1016/j.ajic.2008.03.002 18538699

[22] Neville SA, Lecordier A, Ziochos H, Chater MJ, Gosbell IB, Maley MW, et al Utility of matrix-assisted laser desorption ionization-time of flight mass spectrometry following introduction for routine laboratory bacterial identification. J Clin Microbiol. 2011;49(8):2980–4. 10.1128/JCM.00431-11 21632894 PMC3147730

[23] Shopsin B, Gomez M, Montgomery SO, Smith DH, Waddington M, Dodge DE, et al Evaluation of protein A gene polymorphic region DNA sequencing for typing of Staphylococcus aureus strains. J Clin Microbiol. 1999;37(11):3556–63. 10523551 10.1128/jcm.37.11.3556-3563.1999 PMC85690

[24] Tenover FC, Arbeit RD, Goering RV, Mickelsen PA, Murray BE, Persing DH, et al Interpreting chromosomal DNA restriction patterns produced by pulsed-field gel electrophoresis: criteria for bacterial strain typing. J Clin Microbiol. 1995;33(9):2233–9. 7494007 10.1128/jcm.33.9.2233-2239.1995 PMC228385

[25] Pal S, Juyal D, Adekhandi S, Sharma M, Prakash R, Sharma N, et al Mobile phones: Reservoirs for the transmission of nosocomial pathogens. Adv Biomed Res. 2015;4:144 10.4103/2277-9175.161553 26322292 PMC4549928

[26] Chang CJ, Chen NC, Lao CK, Huang YC. Nasal Staphylococcus aureus and Methicillin-Resistant S. aureus Carriage among Janitors Working in Hospitals in Northern Taiwan. PLoS One. 2015;10(9):e0138971 10.1371/journal.pone.0138971 26407070 PMC4583260

[27] Zhang J, Gu FF, Zhao SY, Xiao SZ, Wang YC, Guo XK, et al Prevalence and Molecular Epidemiology of Staphylococcus aureus among Residents of Seven Nursing Homes in Shanghai. PLoS One. 2015;10(9):e0137593 10.1371/journal.pone.0137593 26340648 PMC4560451

[28] Talwar A, Saxena S, Kumar A. Screening for detection of methicillin-resistant Staphylococcus aureus in Doon Valley Hospitals, Uttarakhand. J Environ Biol. 2016;37(2):247–51. 27097444

[29] Huang YC, Chen CJ. Community-associated meticillin-resistant Staphylococcus aureus in children in Taiwan, 2000s. Int J Antimicrob Agents. 2011;38(1):2–8. 10.1016/j.ijantimicag.2011.01.011 21397461

[30] Huang YC, Su LH, Lin TY. Nasal carriage of methicillin-resistant Staphylococcus aureus among pediatricians in Taiwan. PLoS One. 2013;8(11):e82472 10.1371/journal.pone.0082472 24303083 PMC3841146

[31] Huang YC, Su LH, Chen CJ, Lin TY. Nasal carriage of methicillin-resistant Staphylococcus aureus in school children without identifiable risk factors in northern taiwan. Pediatr Infect Dis J. 2005;24(3):276–8. 15750471 10.1097/01.inf.0000154333.46032.0f

[32] Harmsen D, Claus H, Witte W, Rothganger J, Claus H, Turnwald D, et al Typing of methicillin-resistant Staphylococcus aureus in a university hospital setting by using novel software for spa repeat determination and database management. J Clin Microbiol. 2003;41(12):5442–8. 10.1128/JCM.41.12.5442-5448.2003 14662923 PMC309029

[33] Mellmann A, Friedrich AW, Rosenkotter N, Rothganger J, Karch H, Reintjes R, et al Automated DNA sequence-based early warning system for the detection of methicillin-resistant Staphylococcus aureus outbreaks. PLoS Med. 2006;3(3):e33 10.1371/journal.pmed.0030033 16396609 PMC1325475

[34] http://spaserver2.ridom.de/index.shtml.

[35] Kloos WE, Bannerman TL. Update on clinical significance of coagulase-negative staphylococci. Clin Microbiol Rev. 1994;7(1):117–40. 8118787 10.1128/cmr.7.1.117 PMC358308

[36] Vuong C, Otto M. Staphylococcus epidermidis infections. Microbes Infect. 2002;4(4):481–9. 11932199 10.1016/s1286-4579(02)01563-0

[37] Van Der Zwet WC, Debets-Ossenkopp YJ, Reinders E, Kapi M, Savelkoul PH, Van Elburg RM, et al Nosocomial spread of a Staphylococcus capitis strain with heteroresistance to vancomycin in a neonatal intensive care unit. J Clin Microbiol. 2002;40(7):2520–5. 10.1128/JCM.40.7.2520-2525.2002 12089273 PMC120592

