# Supplementary material for: Nasal colonization and bacterial contamination of mobile phones carried by medical staff in the operating room
Source: PLoS One. 2017 May 31;12(5):e0175811. doi: 10.1371/journal.pone.0175811 (PMC5450997; doi:10.1371/journal.pone.0175811)
Supplement: S1 File — (DOCX) [file pone.0175811.s001.docx]

Supplement 1

Repeat Succession of *Staphylococcus aureus*

S1: 07-23-21-21-17

S2: 15-21-21-477

S3: 08-16-34-282

S4: 233-31-25-17-16-16-16-16

S5: 299-31-25-17-17-16-25-16-111

S6: 7-23-12-12-331

S7: 07-23-12-12-12-21-24-34

S8: 15-21

S9: 07-23-12-12-12-21-24-34

S10: 259-366-23-368-17-16

S11: 07-16-12-23-02-12-23-23-02-34

S12: 07-23-12-21-24-34
